# Clinical Characteristics and Outcomes of Patients Hospitalized for COVID-19 in Africa: Early Insights from the Democratic Republic of the Congo

**DOI:** 10.4269/ajtmh.20-1240

**Published:** 2020-10-02

**Authors:** Jean B. Nachega, Daniel Katuashi Ishoso, John Otshudiema Otokoye, Michel P. Hermans, Rhoderick Neri Machekano, Nadia A. Sam-Agudu, Christian Bongo-Pasi Nswe, Placide Mbala-Kingebeni, Joule Ntwan Madinga, Stéphane Mukendi, Marie Claire Kolié, Edith N. Nkwembe, Gisele M. Mbuyi, Justus M. Nsio, Didier Mukeba Tshialala, Michel Tshiasuma Pipo, Steve Ahuka-Mundeke, Jean-Jacques Muyembe-Tamfum, Lynne Mofenson, Gerald Smith, Edward J. Mills, John W. Mellors, Alimuddin Zumla, Don Jethro Mavungu Landu, Jean-Marie Kayembe

**Affiliations:** 1 Department of Medicine, Centre for Infectious Diseases, Faculty of Medicine and Health Sciences, Stellenbosch University, Cape Town, South Africa;; 2 Department of Epidemiology and International Health, Johns Hopkins Bloomberg School of Public Health, Baltimore, Maryland;; 3 Department of Epidemiology, Infectious Diseases and Microbiology, Center for Global Health, University of Pittsburgh, Pittsburgh, Pennsylvania;; 4 Community Health Department, Kinshasa School of Public Health, University of Kinshasa, Kinshasa, Democratic Republic of the Congo;; 5 Epidemiological Surveillance Team, COVID-19 Response, Health Emergencies Program, World Health Organization, Kinshasa, Democratic Republic of the Congo;; 6 Department of Endocrinology and Nutrition, Cliniques Universitaires St-Luc, Brussels, Belgium;; 7 African Center of Biostatistics Excellence (ACBE), Faculty of Medicine and Health Sciences, Stellenbosch University, Cape Town, South Africa;; 8 Department of Pediatrics, Institute of Human Virology, University of Maryland School of Medicine, Baltimore, Maryland;; 9 International Research Center of Excellence, Institute of Human Virology Nigeria, Abuja, Nigeria;; 10 Department of Paediatrics, University of Cape Coast School of Medical Sciences, Cape Coast, Ghana;; 11 Department of Public Health, Centre Interdisciplinaire de Recherche en Ethnopharmacologie, Faculty of Medicine, Université Notre-Dame du Kasayi, Kananga, Democratic Republic of the Congo;; 12 Faculty of Public Health, Université Moderne de Kinkole, Kinshasa, Democratic Republic of the Congo;; 13 Department of Medical Microbiology and Virology, Faculty of Medicine, University of Kinshasa, National Institute of Biomedical Research (INRB), Kinshasa, Democratic Republic of the Congo;; 14 Direction Surveillance Épidémiologique (DSE), Direction Générale de Lutte contre la Maladie (DGLM), Ministère de la Santé Publique et Riposte COVID-19, Kinshasa, Democratic Republic of the Congo;; 15 Faculty of Medicine, University of Mbuji-Mayi, Mbuji-Mayi, Democratic Republic of the Congo;; 16 Elizabeth Glaser Pediatric AIDS Foundation, Washington, District of Columbia;; 17 Department of Real World & Advanced Analytics, Cytel, Vancouver, Canada;; 18 Department of Health Research Methods, Evidence, and Impact, McMaster University, Hamilton, Canada;; 19 Division of Infectious Diseases, Department of Medicine, University of Pittsburgh, School of Medicine, Pittsburgh, Pennsylvania;; 20 Division of Infection and Immunity, Centre for Clinical Microbiology, University College London, London, United Kingdom;; 21 National Institute for Health Research Biomedical Research Centre, University College London Hospitals NHS Foundation Trust, London, United Kingdom;; 22 Department of Internal Medicine, School of Medicine, University of Kinshasa, Kinshasa, Democratic Republic of Congo

## Abstract

Little is known about the clinical features and outcomes of SARS-CoV-2 infection in Africa. We conducted a retrospective cohort study of patients hospitalized for COVID-19 between March 10, 2020 and July 31, 2020 at seven hospitals in Kinshasa, Democratic Republic of the Congo (DRC). Outcomes included clinical improvement within 30 days (primary) and in-hospital mortality (secondary). Of 766 confirmed COVID-19 cases, 500 (65.6%) were male, with a median (interquartile range [IQR]) age of 46 (34–58) years. One hundred ninety-one (25%) patients had severe/critical disease requiring admission in the intensive care unit (ICU). Six hundred twenty patients (80.9%) improved and were discharged within 30 days of admission. Overall in-hospital mortality was 13.2% (95% CI: 10.9–15.8), and almost 50% among those in the ICU. Independent risk factors for death were age < 20 years (adjusted hazard ratio [aHR] = 6.62, 95% CI: 1.85–23.64), 40–59 years (aHR = 4.45, 95% CI: 1.83–10.79), and ≥ 60 years (aHR = 13.63, 95% CI: 5.70–32.60) compared with those aged 20–39 years, with obesity (aHR = 2.30, 95% CI: 1.24–4.27), and with chronic kidney disease (aHR = 5.33, 95% CI: 1.85–15.35). In marginal structural model analysis, there was no statistically significant difference in odds of clinical improvement (adjusted odds ratio [aOR] = 1.53, 95% CI: 0.88–2.67, *P* = 0.132) nor risk of death (aOR = 0.65, 95% CI: 0.35–1.20) when comparing the use of chloroquine/azithromycin versus other treatments. In this DRC study, the high mortality among patients aged < 20 years and with severe/critical disease is of great concern, and requires further research for confirmation and targeted interventions.

## INTRODUCTION

SARS-CoV-2 infection and COVID-19 arrived later in sub-Saharan Africa (SSA) than in most other regions of the world. As of August 26, 2020, there were 1,014,834 cases and 20,787 deaths (2.1% case fatality rate [CFR]) in the WHO African Region.^1^ The high numbers of cases and deaths expected in SSA have not been witnessed to date, despite relatively weak health systems and other barriers limiting comprehensive implementation of public health interventions.^2^ Several explanations have been hypothesized for this unexpected finding, including early lockdowns, low SARS-CoV-2 testing capacity, a younger population, and concomitant cross-immunity from parasitic diseases and other circulating coronaviruses.^3–6^

The Democratic Republic of the Congo (DRC) confirmed its first COVID-19 case on March 10, 2020 and within 2 weeks declared a state of emergency that included travel bans, lockdowns, widespread testing, and quarantine.^6^ As of August 26, 2020, the DRC has reported 9,891 COVID-19 cases and 251 deaths (2.5% CFR), with the capital city Kinshasa being the epicenter. With increased testing, more COVID-19 cases are being reported in SSA,^3–5^ but data on sociodemographic/clinical characteristics and outcomes among hospitalized patients are still scanty. It is important to ascertain whether features of COVID-19 in Africa differ from those in non-African countries.^7,8^ Furthermore, in SSA, there are little data on the prevalence of SARS-CoV-2 coinfection or comorbidity with noncommunicable diseases (NCDs) (e.g., hypertension, diabetes, and obesity) and communicable diseases (e.g., HIV, tuberculosis [TB], and malaria), which may influence COVID-19 presentations and outcomes.^9,10^ We aimed to describe clinical characteristics, laboratory features, and outcomes of hospitalized patients with COVID-19 in DRC and to differentiate them from other non-African populations.

## METHODS

### Study design, study population, and criteria for hospital admission.

We conducted a cohort analysis using routinely collected data from the DRC Ministry of Health’s COVID-19 Multi-Sectoral Response Committee database, spanning March 10, 2020–July 31, 2020. All COVID-19 patients admitted at the seven largest health facilities in Kinshasa (one private, two faith-based Catholic, and four public) were eligible for inclusion. Patients were staged according to the WHO COVID-19 clinical categories of mild, moderate, severe, and critical disease (Supplemental Table 1).^11^ The decision to hospitalize patients was based on signs or symptoms of moderate/severe disease, comorbidities, pregnancy, or the development of complications in cases initially managed at home.

### Predictors and outcomes variables.

Using standardized data collection forms, we extracted sociodemographic, clinical (including comorbidities), laboratory, COVID-19 treatment, and current medication data. Outcomes of interest were clinical improvement within 30 days (primary) and in-hospital mortality (secondary).

### SARS-CoV-2 RT-PCR testing.

Oropharyngeal or nasal samples were processed at the Virology Laboratory of the National Institute for Biomedical Research in Kinshasa. Samples were tested for SARS-CoV-2 RNA by either BGI RT-PCR using the ABI 7500 Fast Applied Biosystems instrument (Thermo Fisher Scientific, Waltham, MA) or Xpert Xpress SARS-CoV-2 using the GeneXpert platform (Cepheid, Sunnyvale, CA), following the manufacturers’ instructions.

### Case management procedures.

On admission, a detailed history, physical examination, including pulse oximetry, was performed. Self-reported HIV and TB status was further corroborated on admission with a review and confirmation of documented medical record information on relevant medications and/or care for these coinfections. The same was carried out for patients self-reporting NCD comorbidities (e.g., hypertension and diabetes). Patients were treated with symptom management, supplemental oxygen, and compassionate treatment protocols according to national guidelines in effect at the time.^12^ Mild cases were treated with hydroxychloroquine (HCQ)/chloroquine (CQ) + azithromycin (AZ), and moderate cases were treated with HCQ/CQ + AZ (Option 1) or lopinavir/ritonavir (LPV/r) (Option 2) + enoxaparin (prophylactic low–molecular weight heparin).^10^ Severe cases were treated with HCQ/CQ + AZ + third-generation cephalosporin + enoxaparin and assisted ventilation (Option 1), or remdesivir + third-generation cephalosporin + enoxaparin + vitamin C and assisted ventilation (Option 2), or HCQ/CQ + (LPV/r) + third-generation cephalosporin + enoxaparin + dexamethasone and assisted ventilation (Option 3).^12^ As of August 24, 2020, remdesivir has not yet been licensed in the DRC, and not all patients received all indicated treatments because of lack of availability.

### Statistical analysis.

We summarized baseline demographic and clinical characteristics using frequencies and proportions by clinical stage at presentation. Continuous variables were summarized using medians (IQR). Chi-square tests were used to compare proportions and Wilcoxon rank-sum tests to compare medians between mild/moderate and severe/critical cases. COVID-19 symptom resolution was assessed by comparing proportions of patients with symptoms at day 1 (day of admission) versus 10 days later, using the chi-square test for marginal homogeneity. Laboratory values were compared at day 1 and day 10 using the Wilcoxon signed-rank test.

We estimated the proportion of patients with clinical improvement, stratified by baseline demographic and clinical characteristics. Factors associated with clinical improvement at *P*-value < 0.1 in unadjusted univariable logistic regression were included in a multivariable logistic regression model to identify independent factors associated with clinical improvement. The strength of the association was expressed as adjusted odds ratios and accompanying 95% CIs. Similarly, we estimated the hazard of death stratified by baseline characteristics and identified factors independently associated with death using Cox regression. The final regression model was performed after the proportionality of hazards assumption was confirmed by a nonsignificant global test and Schoenfeld residuals with horizontal tendency, as well as the presence of parallelism in the −In[−In(S(t))] plot.

We summarized the strength of association between factors and death using adjusted hazard ratios and associated 95% CIs. We used a marginal structural model (MSM) based on inverse probability of treatment weighting (IPTW) to assess the efficacy of the CQ + AZ combination versus other therapy, with death as the outcome. Gender, age, WHO stage of disease at admission, hypertension, diabetes mellitus, asthma/chronic obstructive pulmonary disease (COPD), heart disease, chronic kidney disease (CKD), HIV, TB, obesity, and cancer were included in the treatment model as potential confounders. All analyses were performed using Stata software version 16.1 (College Station, TX). The Venn diagram illustrations of comorbidities and their combination were completed in R Studio Version 1.3.959, May 2020 (R Studio Inc., Boston, MA).^13^

### Regulatory approvals.

The study was approved by the University of Kinshasa School of Public Health’s Ethics Committee (N°ESP/CE/114/2020 – July 17, 2020); the Institutional Review Board of the University of Pittsburgh, PA (STUDY20080174); and the DRC’s National COVID-19 Multi-Sectorial Response Committee and National Institute of Biomedical Research.

## RESULTS

### Sociodemographic and clinical characteristics of hospitalized confirmed cases.

**Figure 1.**
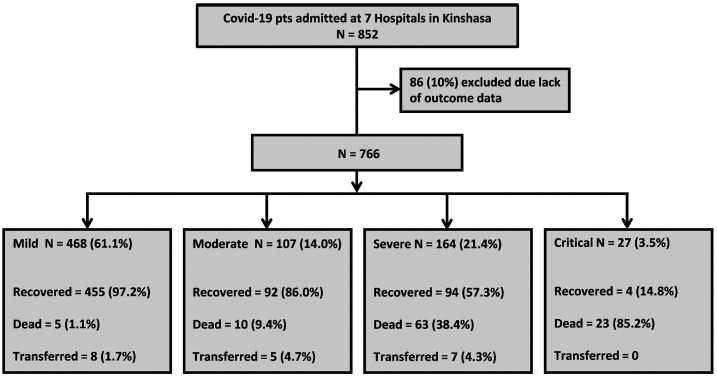
Study flow chart.

Of 852 confirmed COVID-19 cases admitted, we analyzed 766 (89.9%) with complete information (Figure 1). Baseline sociodemographic characteristics and clinical stage did not differ between patients who were excluded and included in the analysis (Supplemental Table 2). Table 1 summarizes patient characteristics at admission by disease severity. The median (IQR) age was 46 (34–58) years, with 23.3% aged ≥ 60 years. Thirty-four (4.5%) patients were < 20 years, with a median age (IQR) of 14.5 (7–18) years and 11 (32%) younger than 10 years. Five hundred (65.3%) patients were male. Among the 262 females admitted, 12 (4.6%) were pregnant. At admission, 468 (61.1%) patients had mild, 107 (14.0%) moderate, 164 (21.4%) severe, and 27 (3.5%) critical disease. All 191 patients with severe or critical disease (25% of total) were admitted to the intensive care unit (ICU). Among 510 patients with SpO_2_ measurements, 38.2% had SpO_2_ ≤ 90% on room air*.* Four of the 34 children presented with severe or critical disease. Compared with those with mild/moderate COVID-19, severe/critical patients had higher median (IQR) C-reactive protein: 60 mg/dL (48.0–192.0) versus 24.0 mg/dL (2.6–54.0), respectively (*P* = 0.010), and median (IQR) D-dimer levels (ng/mL): 342.5 (246.0–443.0) versus 6.9 (2.8–100.0), respectively (*P* = 0.011).

**Table 1:** Demographics and clinical and laboratory characteristics (*N* = 766)

Characteristic	All patients (*n* = 766)	Severe patients (severe and critical) (*n* = 191)	Non-severe patients (mild and moderate) (*n* = 575)	*P*-value
Age median (years) (IQR)	46 (34–58)	58 (44–66)	42 (32–54)	< 0.001
Age-group (years), *n* (%)				
< 20	34 (4.5)	4 (2.1)	30 (5.2)	< 0.001
20–39	248 (32.5)	31 (16.2)	217 (37.9)	–
40–59	303 (39.7)	67 (35.1)	236 (41.3)	–
≥ 60	178 (23.3)	89 (46.6)	89 (15.6)	–
Gender, *n* (%)				
Male	500 (65.6)	135 (71.1)	365 (63.8)	0.078
Female	262 (34.4)	55 (28.9)	207 (36.2)	–
Missing	4	1	3	–
Hypertension, *n* (%)				
Yes	194 (25.4)	87 (45.6)	107 (18.7)	< 0.001
No	570 (74.6)	104 (54.4)	466 (81.3)	–
Missing	2	0	2	–
Heart disease, *n* (%)				
Yes	30 (3.9)	21 (11.1)	9 (1.6)	< 0.001
No	733 (96.1)	169 (88.9)	564 (98.4)	–
Missing	3	1	2	–
Obesity, *n* (%)				
Yes	39 (5.1)	22 (11.5)	17 (3.0)	< 0.001
No	725 (94.9)	169 (88.5)	556 (97.0)	–
Missing	2	0	2	–
Diabetes, *n* (%)				
Yes	107 (14.0)	60 (31.6)	47 (8.2)	< 0.001
No	656 (86.0)	130 (68.4)	526 (91.8)	–
Missing	3	1	2	–
Asthma/chronic obstructive pulmonary disease, *n* (%)				
Yes	26 (3.4)	12 (6.3)	14 (2.4)	0.011
No	738 (96.6)	179 (93.7)	559 (97.6)	–
Missing	2	0	2	–
Chronic kidney disease, *n* (%)				
Yes	7 (0.9)	3 (1.6)	4 (0.7)	0.375
No	759 (99.1)	188 (98.4)	571 (99.3)	–
Cancer, *n* (%)				
Yes	5 (0.6)	3 (1.6)	2 (0.4)	0.102
No	761 (99.4)	188 (98.4)	573 (99.6)	–
Pregnancy among females, *n* (%)				
Yes	12 (4.6)	3 (5.4)	9 (4.4)	0.720
No	250 (95.4)	52 (94.6)	198 (95.6)	–
SpO_2_, *n* (%)				
< 90%	195 (38.2)	166 (92.2)	29 (8.8)	< 0.001
≥ 90%	315 (61.8)	14 (7.8)	301 (91.2)	–
Missing	256	11	245	–
HIV positive, *n* (%)				
Yes	12 (1.6)	3 (1.6)	9 (1.6)	1.000
No	752 (98.4)	188 (98.4)	564 (98.4)	–
Missing	2	0	2	–
Current tuberculosis, *n* (%)				
Yes	19 (2.5)	4 (2.1)	15 (2.6)	0.795
No	745 (97.5)	187 (97.9)	558 (97.4)	–
Missing	2	0	2	–
SpO_2_ (median, IQR), *N*	89.0 (85–98) 510	79 (66–87) 180	98(95–99) 330	< 0.001
Blood glucose (median, IQR) (mg/dL), *N*	105 (23–182) 33	25 (14.5–167.5) 16	131 (103–182) 17	0.031
Serum C-reactive protein (median, IQR) (mg/dL), *N*	32 (3.3–60) 37	60 (48–192) 7	24 (2.6–54) 30	0.010
Serum potassium (median, IQR) (mEq/L), *N*	3.9 (3.4–4.3) 17	4.3 (2.9–4.8) 3	3.9 (3.4–4.0) 14	0.488
Blood urea nitrogen, median (mg/dL), *N*	32.5 (21.0–52.0) 46	49.7 (41.0–63.0) 14	23.1 (20.0–42.2) 32	0.002
Serum creatinine, (mg/dL), *N*	1.0 (0.9–1.2) 48	1.2 (1.0–2.0) 13	1.0 (0.8–1.1) 35	0.008
Plasma D-dimer (median, IQR) (ng/mL), *N*	183 (6.87–349) 11	342.5 (246–443) 6	6.9 (2.8–100) 5	0.011
Electrocardiogram, *n* (%)				
Normal	15 (20.6)	1 (2.7)	14 (38.9)	< 0.001
Abnormal	58 (79.4)	36 (97.3)	22 (61.1)	–
Missing	693	154	539	–
Chloroquine + azithromycin	630 (86.8)	152 (80.8)	478 (88.8)	0.005
Other*	96 (13.2)	36 (19.2)	60 (11.2)	–
Missing	24	2	4	–

* Third-generation cephalosporin and/or amoxicillin +clavulanic acid and/or lopinavir/ritonavir and/or dexamethasone and/or azithromycin.

**Figure 2.**
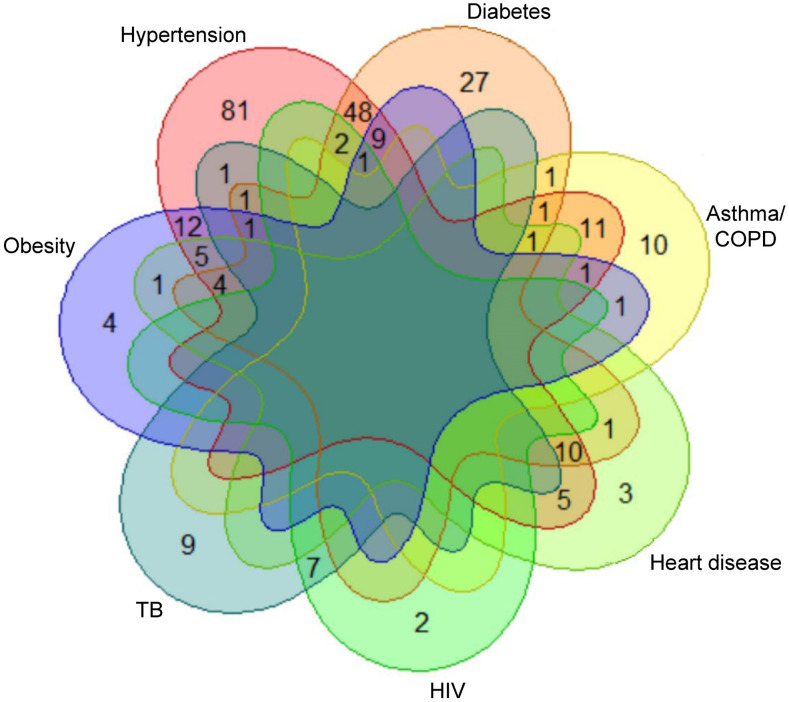
Venn diagram showing overlapping between the main comorbidities among COVID-19 hospitalized patients. Patients with chronic kidney disease (CKD) (*n* = 7) and those with cancer (*n* = 4) were not included in the Venn diagram because of the limitation of the package for a maximum of seven comorbidities. Of the seven patients with CKD, three had concomitant hypertension and diabetes (*n* = 3), DM (*n* = 3), and HTN (*n* = 1). Among the four patients with cancer, one had concomitant heart disease.

Among 764 patients with baseline comorbidity information, 264 (34.6%) reported at least one comorbidity, with 128 (48.5%) having more than one comorbidity (Figure 2). The most prevalent comorbidities were hypertension (25.4%) and diabetes (14.0%). Self-reported prevalence of obesity was 5.1%, heart disease 3.9%, asthma/COPD 3.4%, CKD 0.9%, active TB 2.5%, and HIV 1.6%. Patients with severe/critical disease were older and had a higher prevalence of hypertension, heart disease, obesity, diabetes, asthma/COPD, and poorer SpO_2_ levels than those with mild/moderate disease (Table 1). The majority of patients (*n* = 630, 86.8%) were treated with CQ/AZ. Eighteen patients of 545 (3.1%) on CQ/AZ versus 1/67 (1.5%) on other regimens reported at least one side effect (*P* = 0.70), including pruritus, skin rash, gastrointestinal upset, palpitations, or bradycardia. Overall, 620 patients (80.9%) improved and were discharged within 30 days; 101 (13.2%) died, and 20 (2.6%) were transferred to home care. Median hospital stay among recovered patients was 13 (IQR: 9–17) days. Of the 12 pregnant women, three presented with severe/critical disease and nine had mild/moderate disease. Five had comorbidities (one hypertension and obesity, one asthma, and three TB). All 12 pregnant women recovered and were discharged in 30 days. Four children (11.8%) died; they were 16-, 17-, 17-, and 19-year-olds. Three of four had severe/critical disease (severe pneumonia), and one had moderate disease (pneumonia) at admission.

### Clinical and respiratory parameters at days 1 and 10 of hospitalization.

Supplemental Table 3 presents the changes in markers of infection among patients with assessments at day 1 and day 10. The proportion reporting headaches (23.2% versus 0.6%, respectively, *P* < 0.001), fever (39.8% versus 0.3%, *P* < 0.001), cough (40.9% versus 1.5%, *P* < 0.001), sore throat (12.0% versus 0.8%, *P* < 0.001), rhinorrhea (9.3% versus 0.1%, *P* < 0.001), and dyspnea (37.8% versus 1.0%, *P* < 0.001) decreased markedly between day 1 and day 10. Median oxygen saturation levels significantly improved from 89% to 98% over the same time interval.

### Factors associated with clinical improvement.

The adjusted model for clinical improvement included age-group, the presence of comorbidities (hypertension, heart disease, diabetes, obesity, CKD, and cancer) and treatment received. Table 2 shows factors independently associated with clinical improvement during the observation time. Patients aged 20–39 years (aOR = 9.40, 95% CI: 4.78–18.52) or 40–59 years (aOR = 2.64, 95% CI: 1.64–4.26) were more likely to improve than patients ≥ 60 years. Patients with obesity (aOR = 0.27, 95% CI: 0.12–0.59) were less likely to improve than nonobese patients. Adjusting for treatment with CQ/AZ and age-group, the odds of clinical improvement among patients with severe/critical COVID-19 was 87% lower than among that with mild/moderate disease (aOR = 0.13, 95% CI: 0.08–0.20). In MSM analysis, there was no statistically significant difference in odds of clinical improvement (aOR = 1.53, 95% CI: 0.88–2.67, *P* = 0.132) when comparing the use of CQ/AZ versus other treatment regimens.

**Table 2:** Logistic regression of factors associated with clinical improvement within 30 days (*N* = 766)

Characteristic	Improved, *n* (%)	Unadjusted odds ratio (95% CI)	Adjusted odds ratio (95% CI)	*P*-value
Gender				
Female (*n* = 262)	211 (80.5)	1	–	–
Male (*n* = 500)	406 (81.2)	1.04 (0.71–1.52)	–	–
Age-group (years)				
< 20 (*n* = 34)	29 (85.3)	3.58 (1.32–9.71)	2.98 (1.05–8.49)	0.041
20–39 (*n* = 248)	233 (94.0)	9.60 (5.25–17.55)	9.40 (4.77–18.52)	< 0.001
40–59 (*n* = 303)	246 (81.9)	2.67 (1.76–4.05)	2.64 (1.64–4.26)	< 0.001
≥ 60 (*n* = 178)	110 (61.8)	1	1	–
Clinical stage at presentation				
Mild or moderate (*n* = 575)	525 (91.3)	1	–	–
Severe or critical (*n* = 191)	95 (49.7)	0.09 (0.06–0.14)	–	–
Hypertension				
No (*n* = 570)	480 (84.2)	1	1	–
Yes (*n* = 194)	139 (71.6)	0.47 (0.32–0.70)	1.28 (0.76–2.18)	0.356
Heart disease				
No (*n* = 733)	600 (81.9)	1	1	–
Yes (*n* = 30)	18 (60.0)	0.33 (0.16–0.71)	0.81 (0.32–2.03)	0.656
Diabetes				
No (*n* = 656)	547 (83.4)	1	1	–
Yes (*n* = 107)	71 (66.4)	0.39 (0.25–0.62)	0.76 (0.43–1.35)	0.351
Obesity				
No (*n* = 725)	600 (82.8)	1	1	–
Yes (*n* = 39)	19 (48.7)	0.20 (0.10–0.38)	0.27 (0.12–0.59)	0.001
Asthma/chronic obstructive pulmonary disease				
No (*n* = 738)	600 (81.3)	1	–	–
Yes (*n* = 26)	19 (73.1)	0.62 (0.26–1.51)	–	–
Chronic kidney disease				
No (*n* = 759)	617 (81.3)	1	1	–
Yes (*n* = 7)	3 (42.9)	0.17 (0.04–0.78)	0.22 (0.04–1.08)	0.063
Cancer				
No (*n* = 761)	618 (81.2)	1	1	–
Yes (*n* = 5)	2 (40)	0.15 (0.02–0.93)	0.38 (0.06–2.50)	0.313
HIV				
No (*n* = 752)	611 (81.2)	1	–	–
Yes (*n* = 12)	8 (66.7)	0.46 (0.14–1.55)	–	–
Current tuberculosis				
No (*n* = 745)	604 (81.1)	1	–	–
Yes (*n* = 19)	15 (79.0)	0.88 (0.29–2.68)	–	–
Chloroquine/azithromycin-based treatment vs. other				
No (*n* = 96)	62 (64.6)	1	1	–
Yes (*n* = 630)	526 (83.5)	2.77 (1.74–4.43)	3.62 (2.12–6.16)	< 0.001
Received oxygen				
No (*n* = 330)	307 (93.0)	1	–	–
Yes (*n* = 245)	137 (55.9)	0.10 (0.06–0.16)	–	–

### Factors associated with in-hospital mortality.

**Figure 3.**
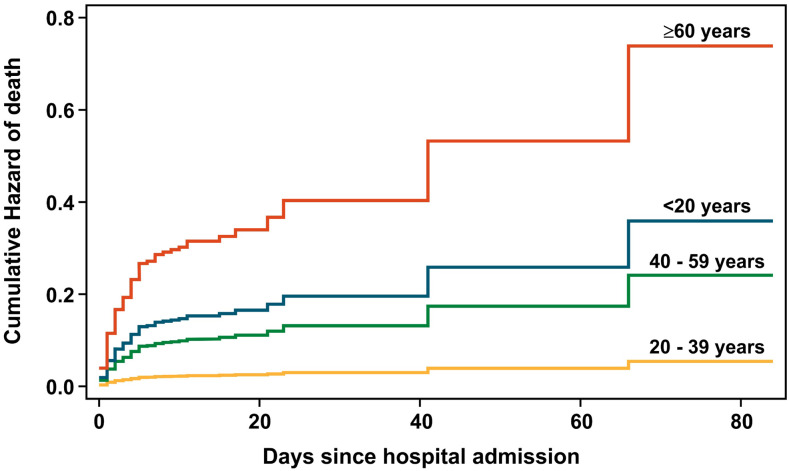
Cumulative hazard of death over time stratified by age-group. The steps in the graph indicate points at which patients died. Patients discharged were censored at time of discharge. The time axis extends to 80 days because that is the longest a patient stayed in hospital.

Overall, in-hospital mortality was 13.2% (95% CI: 10.9–15.8). The median time between admission and death was 4 days (IQR: 2–5). There were no significant gender differences in mortality (13.0% females versus 13.4% males). More patients aged ≥ 60 years (32.0%) died, compared with those < 60 years (7.5%) (*P* < 0.001) (Table 3, Figure 3). In-hospital mortality was greater among patients with severe/critical disease than patients with mild/moderate disease (45.0% versus 2.6%, respectively, *P* < 0.001). Patients < 20 years (aHR = 6.62, 95% CI: 1.85–23.64), 40–59 years (aHR = 4.45, 95% CI: 1.83–10.79), and ≥ 60 years (aHR = 13.63, 95% CI: 5.70–32.60) had significantly higher hazards of death than those aged 20–39 years. Significantly more patients with comorbidities died than those without comorbidities. Among the four children who died, one had diabetes and hypertension and the rest had no comorbidities. Mortality among patients with diabetes was greater than nondiabetics (27.1% versus 10.8%, respectively, *P* < 0.001). More obese versus nonobese patients died (43.6% versus 11.4%, *P* < 0.001). The hazard of death among obese patients was more than double than that for nonobese patients (aHR = 2.30, 95% CI: 1.24–4.27). Compared with those without CKD, patients with CKD were at a higher risk of death (57.1% versus 12.8, *P* < 0.001), with a more than 5-fold increase in the hazard of death (aHR = 5.33, 95% CI: 1.85–15.35). Patients who received CQ/AZ had significantly lower mortality than those who did not receive these drugs (11.0% versus 29.2%, respectively, *P* < 0.001). Mortality in patients receiving supplemental oxygen was greater than that among those who did not (37.6% versus 2.1%, respectively, *P* < 0.001). Patients who received CQ/AZ had a 74% reduction in hazard of death compared with those who did not receive CQ/AZ (aHR = 0.26, 95% CI: 0.16–0.42). However, in MSM analysis, there was no statistically significant difference in risk of death (aOR = 0.65, 95% CI: 0.35–1.20, *P* = 0.166) when comparing use of CQ/AZ versus other treatment regimens.

**Table 3:** Cox regression of factors associated with hazard of death (*N* = 766)

Characteristic	Died, *n* (%)	Unadjusted hazards ratio (95% CI)	Adjusted hazards ratio (95% CI)*	*P*-value
Gender				
Female (*n* = 262)	34 (13.0)	1	–	–
Male (*n* = 500)	67 (13.4)	1.03 (0.68–1.56)	–	–
Age-group (years)				
< 20 (*n* = 34)	4 (11.8)	5.10 (1.44–18.09)	6.62 (1.85–23.65)	0.004
20–39 (*n* = 248)	6 (2.4)	1	1	–
40–59 (*n* = 303)	34 (11.2)	4.62 (1.94–11.01)	4.45 (1.83–10.79)	0.001
≥ 60 (*n* = 178)	57 (32.0)	14.85 (6.40–34.46)	13.63 (5.70–32.60)	< 0.001
Clinical stage at admission				
Mild or moderate (*n* = 575)	15 (2.6)	1	–	–
Severe or critical (*n* = 191)	86 (45.0)	20.84 (12.02–36.14)	–	–
Hypertension				
No (*n* = 570)	56 (9.8)	1	1	–
Yes (*n* = 194)	44 (22.7)	2.32 (1.56–3.45)	1.00 (0.62–1.61)	0.986
Heart disease				
No (*n* = 733)	89 (12.1)	1	1	–
Yes (*n* = 30)	11 (36.7)	3.52 (1.88–6.60)	1.40 (0.68–2.88)	0.364
Diabetes				
No (*n* = 656)	71 (10.8)	1	1	–
Yes (*n* = 107)	29 (27.1)	2.53 (1.64–3.91)	1.10 (0.66–1.81)	0.720
Obesity				
No (*n* = 725)	83 (11.4)	1	1	–
Yes (*n* = 39)	17 (43.6)	3.87 (2.86–6.56)	2.30 (1.24–4.27)	0.009
Asthma/chronic obstructive pulmonary disease				
No (*n* = 738)	96 (13.0)	1	–	–
Yes (*n* = 26)	4 (15.4)	1.27 (0.46–3.45)	–	–
Chronic kidney disease				
No (*n* = 759)	97 (12.8)	1	1	–
Yes (*n* = 7)	4 (57.1)	5.33 (1.96–14.52)	5.33 (1.85–15.35)	0.002
Cancer				
No (*n* = 761)	99 (13.0)	1	–	
Yes (*n* = 5)	2 (40.0)	3.90 (0.96–15.82)	–	–
HIV				
No (*n* = 752)	98 (13.0)	1	–	–
Yes (*n* = 12)	2 (16.7)	1.23 (0.30–4.99)	–	–
Current tuberculosis				
No (*n* = 745)	98 (13.2)	1	–	–
Yes (*n* = 19)	2 (10.5)	0.73 (0.18–2.98)	–	–
Chloroquine/azithromycin–based treatment				
No (*n* = 96)	28 (29.2)	1	1	–
Yes (*n* = 630)	69 (11.0)	0.33 (0.21–0.52)	0.26 (0.16–0.42)	< 0.001
Received oxygen				
No (*n* = 330)	7 (2.1)	1	–	–
Yes (*n* = 245)	92 (37.6)	21.88 (10.14–47.25)	–	–

## DISCUSSION

This study is among the first to report clinical characteristics and outcomes of hospitalized COVID-19 patients in an African country. In this hospitalized Congolese cohort, ∼4.5% of patients were children < 20 years, which is similar to studies from China,^14^ Europe,^15^ and the United States^16^ that have reported between 1% and 5% of infections in children. Given that SARS-CoV-2 testing is more frequently prompted by symptoms and children typically have asymptomatic or mild infection, the frequency of SARS-CoV-2 infection in Congolese children is likely to be higher than 5%.^17^ Similar to Asian and Western cohorts, we observed male gender preponderance and previously reported presenting symptoms, including cough, fever, dyspnea, headache, sore throat, and rhinorrhea.^18^ Age and cardiometabolic comorbidities were associated with more severe forms of COVID-19 at admission and a higher risk of death. Unlike other reports, anosmia and dysgeusia were not documented in our cohort. Not surprisingly, patients admitted with severe COVID-19 were more likely to require oxygen therapy; these patients also differed from those with milder COVID-19 in terms of higher levels of inflammatory markers.

In-hospital mortality was 13.2% in our study population. Global estimates of in-hospital mortality from COVID-19 range between 15% and 20%, with up to 40% of hospitalized patients requiring intensive care.^18^ In Western countries, people of African descent and other racial minorities are at increased risk of worse clinical outcomes.^19^ In a recent U.S. cohort, age and proportion of inpatients with comorbidities were higher than our those in the Congolese cohort: mean age: 54 versus 48 years; hypertension: 44% versus 30%; diabetes: 39% versus 16%; obesity: 35% versus 3.8%; respectively.^20^ Furthermore, our overall in-hospital mortality rate (13.2%) may have been influenced by hospitalization of patients with mild disease who may been admitted because of inadequate care and isolation at home due to overcrowding and/or poverty. However, in-hospital mortality was greater among patients with severe/critical disease than among patients those with mild/moderate disease (45.0% versus 2.6%, respectively, *P* < 0.001), which is higher than Western reports^20^ but similar to the ∼50% mortality of patients requiring admission to the ICU in a South African cohort.^21^ Of note, dexamethasone has recently been shown to reduce mortality by one-third among seriously ill COVID-19 patients requiring oxygen or respiratory support. The drug was introduced in the DRC’s national COVID-19 treatment guidelines^12^ only from July 2020 (last month of our study period), soon after the U.K. Recovery Trial Press Release.^22^ Therefore, further evaluation is needed to ascertain whether in-ICU mortality will decrease with the use of dexamethasone in the DRC.

Among the comorbidities evaluated, hypertension and diabetes were clearly associated with more severe presentation and poorer prognosis for COVID-19. This is in line with findings published from China, the United States, and Europe.^14–16^ These two comorbidities were strongly co-prevalent in our cohort, with 38% of hypertensive patients being diabetic, and 70% of diabetics being hypertensive. More importantly, despite the overall prevalence of self-reported obesity being low (potentially conservative bias due to underestimation), obesity was a significant independent predictor of mortality. Early studies suggest that cytokine release is central to the development of COVID-19–related respiratory distress,^20^ that interleukin-6 (IL-6) is produced by multiple cells including adipocytes,^23,24^ and that IL-6 levels are elevated in obese individuals.^25,26^ Furthermore, adipose tissue has been hypothesized to be a site for SARS-CoV-2 replication and shedding.^27^

In the French Coronavirus SARS-CoV-2 and Diabetes Outcomes (CORONADO) study, among diabetic inpatients with COVID-19, body mass index and poor long-term glucose control were independently associated with mechanical ventilation and/or death.^28^ Several arguments suggest that there is no causal link between severe pneumonia and chronic hyperglycemia and that the overrepresentation of diabetic patients with COVID-19 in ICUs indirectly reflects the impact of obesity.^29^ Furthermore, higher hemoglobin A1c (HbA1c) at admission does not appear to worsen COVID-19 prognosis in type II diabetes.^30^ For this study, there were no HbA1c data available; thus, we were unable to analyze its potential association with COVID-19 outcomes. We also found that CKD was an independent risk factor for mortality, as reported from outside Africa.^31–34^ Patients presenting with SARS-CoV-2 infection have shown varying degrees of renal dysfunction, including a high incidence of acute kidney injury.^32,35^ A recent study reported that the human kidney may be a unique target for SARS-CoV-2 because it expresses angiotensin-converting enzyme-2 surface receptors.^32,33,35^

There was no significant difference in mortality when comparing CQ/AZ versus other regimens by MSM analysis. Our data do not conclusively exclude a CQ/AZ treatment effect, given the lack of a comparator arm, and nonrandom treatment allocation. However, recently published placebo-controlled trials from the United States,^36^ the United Kingdom,^37^ and Brazil^38^ have shown no effect of CQ or CQ/AZ on COVID-19 mortality. There is an urgent need for rigorous evaluation of other promising and scalable cost-effective therapeutic options for the DRC and other African countries.

Our findings showed no association of HIV and/or TB with baseline COVID-19 disease severity or prognosis. However, definitive conclusions cannot be made because of low prevalence of HIV (1.6%) and active TB (2.2%) in our cohort. A population-based U.K. study found that HIV-positive individuals had more than double the risk of COVID-19–related mortality than HIV-uninfected individuals after controlling for known confounding factors.^39^ Similarly, a retrospective analysis of > 20,000 South African adults with COVID-19 showed that HIV was associated with a doubling of COVID-19 mortality risk, although this may be an overestimate because of residual confounders.^40^ Larger SSA cohort studies are required to further define the epidemiological, clinical, and risk relationships among the overlapping epidemics of COVID-19, HIV, TB, and malaria.

Of great concern is that children and adolescents < 20 years had a CFR of 11.8% and were nearly seven times more likely to die than patients aged 20–39 years. By contrast, studies mostly from China report CFRs of < 1% among both symptomatic and asymptomatic, but mostly hospitalized children.^41,42^ A recent U.S. study reported a CFR of 2% among children hospitalized with COVID-19).^43^ All four pediatric deaths in our DRC cohort occurred among older children aged 16–19 years. Three of the four had severe/critical disease, and one had moderate disease at admission; also, three of these four cases had no underlying comorbidity. Webb et al.^44^ recently reported on 23 South African children with MIS-C, among whom 52% required ICU admission primarily because of cardiac dysfunction. There were no deaths reported in this South African cohort; all the children survived. Multisystem inflammatory syndrome was not specifically reported in our DRC pediatric cohort, but it may not have been recognized. Our study’s small number of children and the possibility of unmeasured confounding factors, such as the availability of necessary equipment, quality, and scope of pediatric intensive care, preclude concrete conclusions about excess COVID-19–related mortality among children and adolescents in the DRC. This calls for larger, robust investigations of COVID-19 outcomes among hospitalized children in SSA.

Our study has some limitations. Approximately 10% of patients had missing data on outcomes of interest and were not included in our analysis. However, these patients were comparable with those included with respect to sociodemographic characteristics and COVID-19 clinical stage. In addition, we were not able to compare clinical characteristics between hospitalized COVID-19 patients and outpatients. Finally, given the low prevalence of self-reported HIV and/or TB status, we cannot speculate on the impact of these conditions on COVID-19 outcomes. Strengths of our study include a robust sample size of hospitalized patients in SSA from where little information on COVID-19 has been reported. We also provide data on 34 children, a population for whom there are even less COVID-19 data available from SSA.^9^ Finally, the use of robust statistical methods such as MSM and IPTW creates more balanced comparisons between treatment groups, similar to those that would be found in a randomized clinical trial.

## CONCLUSION

In this study, hospitalized patients in SSA with COVID-19 had a somewhat lower overall in-hospital mortality than hospitalized patients in non-African regions, but mortality in those with severe or critical disease was almost 50%. Age-groups at high risk and comorbidities associated with death were similar between our cohort and those from prior studies in Asia, Europe, and North America. Although our study provides insights into COVID-19 manifestations in Africa, more data are needed from countries across this region. Large-cohort observational studies are required to better define the epidemiology and factors affecting COVID-19 outcomes and the relationships between the overlapping epidemics of COVID-19, HIV, TB, and malaria among both young and older populations. Rigorous evaluations of promising, scalable, and cost-effective therapeutics and vaccines are needed globally.

## Supplemental tables

Supplemental materials
