# Supplementary material for: Clinical Characteristics and Outcomes of Patients Hospitalized for COVID-19 in Africa: Early Insights from the Democratic Republic of the Congo
Source: Am J Trop Med Hyg. 2020 Oct 2;103(6):2419–28. doi: 10.4269/ajtmh.20-1240 (PMC7695108; doi:10.4269/ajtmh.20-1240)
Supplement: Supplementary file 1 [file tpmd201240.SD1.docx]

| **Supplemental Table 1. World Health Organization COVID-19 disease severity** | | |
| --- | --- | --- |
|  |  | |
| **Mild disease** | Symptomatic patients meeting the case definition for  COVID-19 without evidence of viral pneumonia or hypoxia | |
| **Moderate disease** |  | Adolescent or adult with clinical signs of pneumonia (fever, cough, dyspnea, fast breathing) but no signs of severe pneumonia, including SpO_2_  ≥ 90% on room air. Child with clinical signs of non-severe pneumonia (cough or difficulty breathing + fast breathing and/or chest indrawing) and no signs of severe pneumonia. Fast breathing (in breaths/min): < 2 months: ≥ 60; 2–11 months: ≥ 50; 1–5 years: ≥ 40. |
|  | **Pneumonia** |  |
| **Severe disease** | **Severe**  **pneumonia** | **Adolescent or adult** with clinical signs of pneumonia (fever, cough, dyspnea, fast breathing) + one of the following: respiratory rate > 30 breaths/min; severe respiratory distress; or SpO2 < 90% on room air.  **Child** with clinical signs of pneumonia (cough or difficulty in  breathing) + at least one of the following:  • Central cyanosis or SpO_2_ < 90%; severe respiratory distress (e.g. fast breathing, grunting, very severe chest indrawing); general danger sign: inability to breastfeed or drink, lethargy or unconsciousness, or convulsions.  • Fast breathing (in breaths/min): < 2 months: ≥ 60; 2–11  months: ≥ 50; 1–5 years: ≥ 40. While the diagnosis can be made on clinical grounds; chest imaging (radiograph, CT scan, ultrasound) may assist in diagnosis and identify or exclude pulmonary complications. |
|  |  |  |
| **Critical disease** | **Acute respiratory**  **distress syndrome (ARDS)** | Onset: within 1 week of a known clinical insult (i.e. pneumonia) or new or worsening respiratory symptoms.  **Chest imaging**: (radiograph, CT scan, or lung ultrasound): bilateral opacities, not fully explained by volume overload, lobar or lung collapse, or nodules.  Origin of **pulmonary infiltrates**: respiratory failure not fully explained by cardiac failure or fluid overload. Need objective assessment (e.g. echocardiography) to exclude hydrostatic cause of infiltrates/oedema if no risk factor present.  **Oxygenation impairment in adults:**  **• Mild ARDS**: 200 mmHg < PaO_2_/FiO_2_  a ≤ 300 mmHg (with PEEP or  CPAP ≥ 5 cmH2O)  • **Moderate ARDS**: 100 mmHg < PaO2/FiO2 ≤ 200 mmHg (with PEEP ≥ 5 cmH2O).  • **Severe ARDS**: PaO2/FiO2 ≤ 100 mmHg (with PEEP ≥ 5 cmH2O).  **Oxygenation impairment in children**: note OI and OSI.c Use OI when available. If PaO2 not available, wean FiO2 to maintain SpO2 ≤ 97% to calculate OSI or SpO2/FiO2 ratio:  • Bilevel (NIV or CPAP) ≥ 5 cmH2O via full face mask:  PaO2/FiO2  ≤ 300 mmHg or SpO2/FiO2 ≤ 264.  • **Mild ARDS** (invasively ventilated): 4 ≤ OI < 8 or 5 ≤ OSI < 7.5.  • **Moderate ARDS** (invasively ventilated): 8 ≤ OI < 16 or 7.5 ≤ OSI< 12.3.  • **Severe ARDS** (invasively ventilated): OI ≥ 16 or OSI ≥ 12.3. |
| **Critical disease** | **Septic choc** | **Adults:** persistent hypotension despite volume resuscitation, requiring vasopressors to maintain MAP ≥ 65 mmHg and serum lactate level > 2 mmol/L.  **Children**: any hypotension (SBP < 5th centile or > 2 SD below normal for age) or two or three of the following: altered mental status; bradycardia or tachycardia (HR < 90 bpm or > 160 bpm in infants and heart rate < 70 bpm or > 150 bpm in children); prolonged capillary refill (> 2 sec) or weak pulse; fast breathing; mottled or cool skin or petechial or purpuric rash; high lactate; reduced urine output; hyperthermia or hypothermia |

**Supplemental Table 2. Characteristics of patients with observed outcomes compared to patients with missing outcomes**

| **Characteristics** |  | | | **P-Value** |
| --- | --- | --- | --- | --- |
|  | **Observed**  **(n =766)** | | **Missing**  **(n =86)** |  |
| Age median (IQR) | 48 (36 - 62) | | 46 (34 -58) | 0.265 |
| Age group n (%) |  | |  |  |
| < 20 years | 34 (4.5) | | 1 (1.2) | 0.076 |
| 20 – 39 years | 248 (32.5) | | 32 (37.6) |  |
| 40 – 59 years | 303 (39.7) | | 25 (29.4) |  |
| >= 60 years | 178 (23.3) | | 27 (31.8) |  |
| Missing | 3 | | 1 |  |
| Sex, n (%) |  | |  |  |
| Male | 500 (65.6) | | 53 (61.6) | 0.462 |
| Female | 262 (34.4) | | 33 (38.4) |  |
| Missing | 4 | | 0 |  |
| Clinical stage at admission |  | |  |  |
| Mild or moderate (n=575) | 575 (75.1) | | 71 (82.6) | 0.124 |
| Severe or critical (n=191) | 191 (24.9) | | 15 (17.4) |  |
| Hypertension, n (%) |  | |  |  |
| Yes | 194 (25.4) | | 22 (27.2) | 0.729 |
| No | 570 (74.6) | | 59 (72.8) |  |
| Missing | 2 | | 5 |  |
| Heart disease, n (%) |  | |  |  |
| Yes | 30 (3.9) | | 4 (4.9) | 0.610 |
| No | 733 (96.1) | | 77 (95.1) |  |
| Missing | 3 | | 5 |  |
| Obesity, n (%) |  | |  |  |
| Yes | 39 (5.1) | | 2 (2.5) | 0.418 |
| No | 725 (94.9) | | 79 (97.5) |  |
| Missing | 2 | | 2 |  |
| Diabetes, n (%) |  | |  |  |
| Yes | 107 (14.0) | | 9 (11.1) | 0.559 |
| No | 656 (86.0) | | 72 (88.9) |  |
| Missing | 1 | | 2 |  |
| Asthma/COPD, n (%) |  | |  |  |
| Yes | 26 (3.4) | | 1 (2.6) | 1.000 |
| No | 738 (96.6) | | 38 (97.4) |  |
| Missing | 2 | | 47 |  |
| CKD, n (%) |  | |  |  |
| Yes | 7 (0.9) | | 0 | 1.000 |
| No | 759 (99.1) | | 86 (100) |  |
| Cancer, n (%) |  | |  |  |
| Yes | 5 (0.6) | | 0 | 1.000 |
| No | 761 (99.4) | | 86 (100) |  |
| SpO_2_, n (%) |  | |  |  |
| < 90% | 195 (38.2) | | 25 (32.9) | 0.370 |
| ≥ 90% | 315 (61.8) | | 51 (67.1) |  |
| Missing | 256 | | 10 |  |
| EKG n **(%)**  Normal | 15 (20.6) | 2 (20.0) | | 1.000 |
| Abnormal | 58 (79.4) | 8 (80.0) | |  |
| Missing | 693 | 76 | |  |
| Chloroquine + Azithromycin | 630 (86.8) | 78 (91.8) | | 0.230 |
| Other* | 96 (13.2) | 7 (8.2) | |  |
| Missing | 40 | 1 | |  |

**Supplemental Table 3. Clinical and respiratory parameters at days 1 and 10 of hospitalization (N = 733)**

| **Symptoms/biological parameters** | **Period** | | **P-Value** |
| --- | --- | --- | --- |
|  | **Day1**  **n (%)** | **Day10**  **n (%)** |  |
| **Headache** |  |  |  |
| Yes | 171 (23.2) | 4 (0.6) | <0.001 |
| No | 562 (76.7) | 729 (99.4) |  |
| **Fever** |  |  |  |
| Yes | 292 (39.8) | 2 (0.3) | <0.001 |
| No | 441 (60.2) | 731 (99.7) |  |
| **Cough** |  |  |  |
| Yes | 300 (40.9) | 11 (1.5) | <0.001 |
| No | 433 (59.1) | 722 (98.5) |  |
| **Sore throat** |  |  |  |
| Yes | 88 (12.0) | 6 (0.8) | <0.001 |
| No | 645 (88.0) | 727 (99.2) |  |
| **Rhinorrhea** |  |  |  |
| Yes | 68 (9.3) | 1 (0.1) | <0.001 |
| No | 665 (90.7) | 732 (99.9) |  |
| **Dyspnea** |  |  |  |
| Yes | 277 (37.8) | 7 (1.0) | <0.001 |
| No | 456 (62.2) | 726 (99.0) |  |
| SpO_2_ (median, IQR) (%) | 96 (87 - 98) | 98 (97 - 99) | <0.001 |
| N=264 |  |  |  |
